# A Full-Profile Measurement Method for an Inner Wall with Narrow-Aperture and Large-Cavity Parts Based on Line-Structured Light Rotary Scanning

**DOI:** 10.3390/s25092843

**Published:** 2025-04-30

**Authors:** Zhengwen Li, Changshuai Fang, Xiaodong Zhang

**Affiliations:** State Key Laboratory of Precision Measuring Technology & Instruments, Laboratory of Micro/Nano Manufacturing Technology, Tianjin University, Tianjin 300072, China; lzw1035@tju.edu.cn (Z.L.); cshfang@tju.edu.cn (C.F.)

**Keywords:** line-structured light, rotary scanning, multi-sensor registration

## Abstract

As a special component, inner-wall-shaped parts with a narrow aperture and large cavity play an important role in the field of industrial manufacturing. It is of great significance to accurately measure the full profile of the inner surface of such parts. Line-structured light scanning is a widely used method for inner wall 3D measurement, which is usually applied to linear scanning measurements of the inner wall of pipe-shaped parts. In view of the structural characteristics of narrow-aperture and large-cavity parts, this article establishes a multi-sensor scanning measurement system based on the principle of line-structured light, which adopts rotary scanning instead of the traditional linear scanning measurement method in the system. Additionally, a calibration method is introduced to resolve the challenges associated with the calibration of rotation axis parameters. Considering the structural constraints in the measurement of narrow-aperture and large-cavity parts, a structural optimization algorithm is designed to enable the sensor to achieve a high theoretical measurement resolution while satisfying the geometric constraints of the measured parts. In order to minimize the size of the sensor, the adjacent sub-sensors in the system are arranged in the form of low overlapping fields of view (FOV). To solve the problem of multi-sensor registration under low overlapping FOV, a calibration method based on the structural characteristics of the measurement system itself is proposed, which realizes low-cost and high-precision calibration of the multi-sensor system. Through the repeatability measurement experiment of the spherical cavity parts, the average measurement deviation of the spherical cavity radius was measured to be 6 μm, and the standard deviation was 11.4 μm, which verified the feasibility of the measurement system proposed in this article. By comparing the system calibration method proposed in this article with existing methods, the measurement accuracy of the system is improved by approximately 80%, demonstrating the effectiveness of the proposed method.

## 1. Introduction

With the rapid development of industrial manufacturing technology, inner-wall-shaped parts are widely used in industrial production, such as engine cylinder housings [1] and pipeline ball valves [2,3]. Among them, an inner-wall-shaped part with a narrow aperture and large cavity is a type of part with special structural and critical functions that plays an irreplaceable role in the field of industrial manufacturing. For example, in the manufacture of precision optical components, the inner wall surface quality of the injection mold will affect the molding accuracy and optical performance of the optical components [4,5]. In addition, such parts are also used as carriers of certain special substances in industrial manufacturing for research in fluid mechanics and explosion characteristics [6,7]. The opening size of such parts is usually in the order of tens of millimeters, while the diameter of the inner cavity can reach hundreds of millimeters. Therefore, it is of great significance to perform a complete measurement of the inner surface 3D profile of such parts to obtain the critical geometric dimensions of structures on their inner walls. However, there are few studies on inner wall 3D measurement methods for narrow-aperture and large-cavity parts, and the special structural characteristics of such parts also bring difficulties to the design of sensors and measurement systems.

For inner-wall-shaped parts, the existing 3D measurement methods are mainly based on optical non-contact measurement methods. Compared with the low efficiency and poor flexibility of contact measurement methods [8], optical measurement methods are widely adopted due to being non-contact, with fast speed and high precision. The measurement form can be divided into three types: point, line, and surface. The point measurement method is limited by measurement efficiency and data density, and its reliance on multi-degree-of-freedom motion mechanisms introduces motion errors into the measurement process, which makes it unsuitable for high-precision and rapid measurement in industrial settings. Wu et al. [9] designed an inner-wall measurement system for pipelines based on a position-sensitive detector, which realized 3D reconstruction of small-diameter pipelines. However, the measurement accuracy of the system for the inner diameter of the pipe is limited to 0.1 mm, indicating insufficient precision. Zhang et al. [10] achieved 3D measurement of the groove structure on the inner surface of ball screw nuts with an inner diameter of 40.5 mm based on spectral confocal technology. The sensor measurement accuracy can achieve 1 μm. However, the confocal measurement principle has problems such as high cost and small measurement range, which makes it unsuitable for the full-profile measurement of the inner wall. There are two main surface measurement methods: fringe projection and digital holography [11]. Masayuki et al. [12] designed a multi-wavelength digital holographic detection system to detect and classify defects on the inner wall of copper tubes. César-Cruz et al. [13] designed a measurement system for 3D reconstruction of cylindrical objects based on the principle of fringe projection. The system can realize 3D reconstruction of objects placed on the inner surface of a cylinder, but the measurement deviation is only on the millimeter scale, resulting in low accuracy. The sensor constructed by the surface measurement principle is relatively complex in structure and imposes higher hardware requirements on the measurement system, which makes it impossible to achieve full-profile measurement of the inner wall of the blind hole structure, limiting the measurement range and flexibility. In contrast, linear measurement methods have the advantages of high measurement accuracy and efficiency. Due to its inherent simplicity in implementation, the line-structured light measurement principle can be applied to a variety of different measurement occasions. This technique has been extensively implemented for dimensional inspection of pipeline and cylinder inner walls [14,15], as well as precision measurement of small bores and slots, with equal effectiveness [16,17].

However, most of the current research on linear measurement methods for inner wall parts employs linear axis scanning to measure structures such as cylindrical inner walls, and the measurement accuracy is limited by the motion precision of the linear axis. In contrast, the rotation axis scanning method is more suitable for 3D measurement of narrow-aperture and large-cavity parts. Additionally, due to the circular closure property of angular indexing devices, the rotation axis can achieve higher motion accuracy compared to the linear axis [18,19]. Therefore, this article adopts a line-structured light rotary scanning method to build the measurement system. In order to avoid the problem in practical situations where the measured object has been fixed and is not suitable for movement, and to reduce the influence of the measured object load on the movement of the rotation axis, the system adopts a self-rotating scanning measurement method where the sensor rotates around the axis. When calibrating the relative position between the rotation axis and the sub-sensor in the self-rotating measurement system, the main challenge lies in the continuous change of the measurement range during rotation. This makes it difficult to calibrate the axis position by constructing feature points around the axis in the sensor coordinate system [20]. In current research on calibration methods for the rotation axis in a self-rotating sensor coordinate system, Niu et al. [21] employed an auxiliary camera and checkerboard to calibrate the rotation axis. They utilized stereo vision principles to determine the relative position between the camera on the rotating platform and the rotation axis. However, this method suffers from cumulative errors introduced by distributed calibration, and it also has limitations due to the fixed position of the auxiliary target. Liu et al. [22] proposed a method where the camera and projector are rotated to different angles using a rotating mechanism to capture point cloud data of the same object from various positions. Then, they performed point cloud registration sequentially to establish the relationship between the camera and the rotation axis. However, this method tends to introduce significant cumulative errors in practical applications. To address this problem, this article proposes a calibration method for a rotation axis based on optimization, where the rotation axis position is optimized to obtain the precise relationship between the sensor and the rotation center by scanning ring gauges at different positions.

In order to achieve full-profile measurement of inner-wall-shaped parts, the measurement system must have a large field of view (FOV). Two solutions are commonly used to address the issue: a multi-axis measurement system and a multi-sensor measurement system [23,24]. Since the inner-wall-shaped parts have the characteristic of a small space, using a multi-axis measurement system will lead to a risk of collision during measurement and increase system complexity. In contrast, the multi-sensor configuration can provide complete coverage of the measurement field and has higher measurement efficiency. For narrow-aperture and large-cavity parts, their structural constraints lead to a contradiction between the measurement accuracy and the geometric parameters of the sensor. Therefore, it is necessary to design the structural parameters of the line-structured light sub-sensor to meet the various constraints present in practical measurements. Currently, many studies have analyzed the impact of parameters in line-structured light systems on measurement accuracy from various perspectives [25,26]. They have proposed many optimization design strategies to provide theoretical guidance for the design of line-structured light sensors [27,28]. However, there is a lack of application to real engineering measurement problems with multiple complex constraints.

To ensure that the sensor size meets the aperture limitations of the parts, this article adopts a configuration of low overlapping FOV for arranging multiple sub-sensors in the multi-sensor measurement system. Precisely determining the relative position among the sub-sensors is critical for achieving high-precision measurements. The key to the multi-sensor registration problem under low overlapping FOV is to construct a global feature. There are mainly two types of methods. One of them uses auxiliary targets to establish the relative position between two sub-sensors [29,30]. Another method uses high-precision 3D equipment such as theodolites or laser trackers to obtain global feature points [31,32]. These methods still have limitations in practice, as they depend on customized large calibration targets, auxiliary equipment, and complex operational procedures. This results in a lack of flexibility and generalizability, as well as higher implementation costs. Moreover, most existing calibration methods primarily focus on establishing multi-camera relationships from the perspective of camera imaging within the sensor, without analyzing the final measurement data generated by the sensor.

To address the limitations of existing measurement methods, this article proposes a multi-sensor self-rotating scanning measurement system based on the principle of line-structured light. This approach overcomes the constraints of traditional methods in addressing the 3D measurement of narrow-aperture and large-cavity parts, providing an innovative solution for the 3D measurement of inner walls with complex structures. The main contributions of this article are as follows:

(1) To address the issues of high measurement costs and low accuracy caused by axis hardware in traditional linear axis scanning measurement systems, this article proposes a self-rotating axis scanning approach to construct the measurement system. This method reduces hardware costs while improving measurement accuracy and efficiency, providing a reference for the design of three-dimensional measurement systems for inner-wall parts. In response to the challenge of rotation axis calibration in self-rotating scanning measurement systems, this article introduces a simple and high-precision method for calibration the self-rotating axis.

(2) Due to the various structural constraints in the full-profile measurement of narrow-aperture and large-cavity parts, there exists a contradiction between the measurement accuracy and the geometric size of the sensor. This article proposes a structural parameters optimization design algorithm to achieve the best theoretical measurement accuracy while satisfying the constraints. It provides a reference solution for structural optimization under geometric constraints.

(3) To address the issues of low accuracy and high calibration costs in existing multi-sensor registration methods under low overlapping FOV, this article proposes a registration method based on the structural constraints of the measurement system itself. It provides a low-cost and high-precision solution for multi-sensor registration in such conditions.

**Figure 1:**
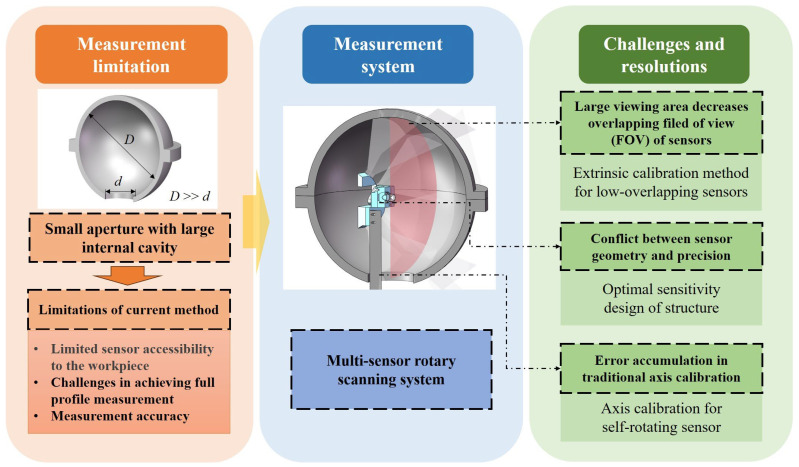
Difficulties and solutions of measuring narrow-aperture and large-cavity parts.

The rest of this article is organized as follows. Section 2 introduces the geometric principle of the measurement system. Section 3 introduces the parameter analysis and optimization strategy for the line-structured light system. Section 4 introduces the rotation axis calibration and multi-sensor registration methods. Section 5 presents the experimental results of this article. Section 6 provides a summary of this article. The technical route of this article is shown in Figure 1.

## 2. Measurement System and Principles

**Figure 2:**
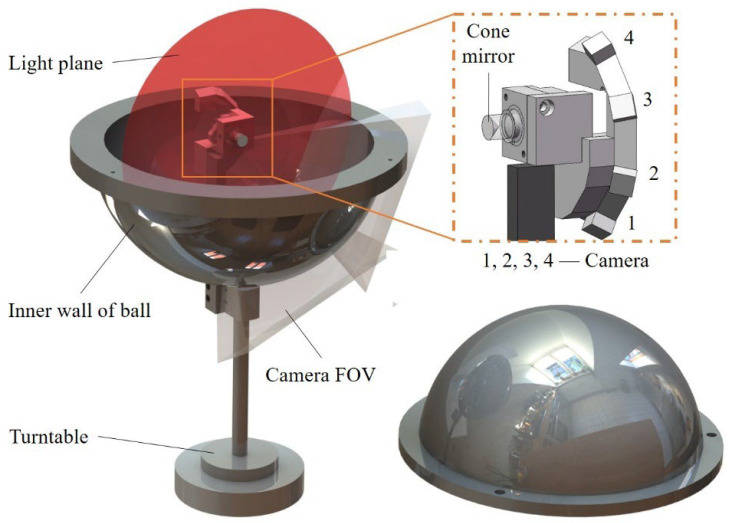
System structure schematic.

This article utilizes spherical inner cavity parts as the test objects for analysis. As shown in Figure 2, the sensor of the measurement system consists of four small cameras and a laser. The light emitted by the laser diode passes through a collimating lens and is directed onto a conical mirror with a 90° apex angle, generating the light plane. This plane intersects with the inner wall of the spherical cavity and produces a circular light stripe. The fields of view of the four cameras collectively cover half of the inner wall area. The full profile of the inner wall of the spherical cavity can be measured by driving the sensor to rotate 360° via a turntable.

Each camera and laser form a sub-sensor, and it follows the laser triangulation principle. As shown in Equation (1), the camera imaging process is regarded as a pinhole model [33] and is combined with the light plane equation.

(1) $$\left\{\begin{array}{c} Z_{c}\left[\begin{array}{c} u\\ v\\ 1 \end{array}\right]=\left[\begin{array}{ccc} F_{x} & 0 & u_{0}\\ 0 & F_{y} & v_{0}\\ 0 & 0 & 1 \end{array}\right]\left[\begin{array}{c} X_{c}\\ Y_{c}\\ Z_{c} \end{array}\right]\\ AX_{c}+BY_{c}+CZ_{c}+D=0 \end{array}\right.$$

In Equation (1), *u* and *v* represent the pixel coordinates of the center of the light strip in the image captured by the camera, and (*X_c_*, *Y_c_*, *Z_c_*) represents the three-dimensional coordinates of the space point corresponding to the center pixel of the light strip in the camera coordinate system. *A*, *B*, *C*, and *D* are the light plane parameters. *F_x_* and *F_y_* are the equivalent focal lengths in the X and Y directions in the camera coordinate system. *u*_0_ and *v*_0_ represent the coordinates of the principal point.

(2) $$\left[\begin{array}{c} X_{w}\\ Y_{w}\\ Z_{w} \end{array}\right]=\cup RT_{i}\left[\begin{array}{c} X_{ci}\\ Y_{ci}\\ Z_{ci} \end{array}\right]$$

The point cloud data of the measured surface can be obtained by integrating the point cloud data of the four sensors, as shown in Equation (2), where (*X_w_*, *Y_w_*, *Z_w_*) represents the point cloud coordinates in the world coordinate system and (*X_ci_*, *Y_ci_*, *Z_ci_*) represents the three-dimensional coordinates in the *i*-th camera coordinate system. *RT_i_* represents the rigid body transformation matrix from the *i*-th camera coordinate system to the world coordinate system.

## 3. Structural Optimization Design of Full-Profile Measurement System Under Aperture Constraints

### 3.1. Precision Analysis of Line-Structured Light Measurement

The measurement accuracy of a line-structured light sensor is influenced by various hardware structural parameters. When addressing practical sensor design requirements, the values of these parameters are often subject to certain constraints. Therefore, it is necessary to analyze the impact of different parameters on measurement accuracy, which guarantees that the designed sensor achieves optimal theoretical measurement accuracy while satisfying external constraints.

**Figure 3:**
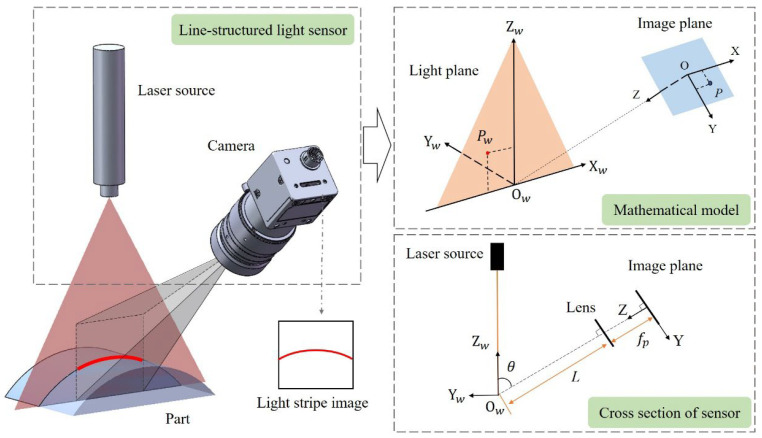
Geometric model of line-structured light vision sensor.

According to the laser triangulation principle, the measurement principle of the line-structured light measurement system is shown in Figure 3. The correspondence between the 2D pixel coordinates in the camera target surface and the 3D spatial coordinates in the light plane is established by calibrating the relative position between the camera and the light plane. The world coordinate system O_w_-X_w_Y_w_Z_w_ is established based on the position of the light plane. O-XYZ is the image coordinate system, and the X-axis in the image coordinate system is parallel to the X_w_-axis in the world coordinate system. *θ* represents the angle between the camera optical axis *OO_w_* and the light plane; *L* represents the working distance of the camera; *f_p_* represents the distance from the optical center of the lens to the imaging plane. According to the geometric relationship, for any point *P_w_* in the light plane, the coordinates of its corresponding point *P* in the imaging plane can be expressed by Equation (3), where *d* represents the pixel size of the camera.

(3) $$\left\{\begin{array}{c} X_{w}=\frac{LX\sin\theta }{f_{p}\sin\theta +Y\cos\theta }\\ Z_{w}=\frac{LY}{f_{p}\sin\theta +Y\cos\theta }\\ X=(u-u_{0})d\\ Y=(v-v_{0})d \end{array}\right.$$

The measurement accuracy of a line-structured light measurement system can be reflected by its measurement resolving power, which is defined as the minimum change in the measured quantity that can cause a detectable change in the indication value. In the line-structured light measurement system, the change of the indicated value can be considered as the positional change of pixels in the imaging plane of the camera. Based on Equation (3), the resolving power of the system in the X_w_ and Z_w_ directions can be obtained by calculating the partial derivatives of *u* and *v*. For the convenience of analysis, the expression for the system’s measurement resolving power *μ* is defined as shown in Equation (4).

(4) $$\left\{\begin{array}{c} \frac{\partial X_{w}}{\partial u}=\frac{Ld\sin\theta }{f_{p}\sin\theta +(v-v_{0})d\cos\theta }\\ \frac{\partial X_{w}}{\partial v}=-\frac{(u-u_{0})Ld^{2}\sin\theta \cos\theta }{{\left[f_{p}\sin\theta +(v-v_{0})d\cos\theta \right]}^{2}}\\ \frac{\partial Z_{w}}{\partial v}=\frac{Lf_{p}d\sin\theta }{{\left[f_{p}\sin\theta +(v-v_{0})d\cos\theta \right]}^{2}}\\ \mu =\frac{1}{2}\sqrt{{\left(\frac{\partial X_{w}}{\partial u}\right)}^{2}+{\left(\frac{\partial X_{w}}{\partial v}\right)}^{2}+{\left(\frac{\partial Z_{w}}{\partial v}\right)}^{2}} \end{array}\right.$$

According to Equation (4), it can be seen that the resolving power of the line-structured light system is related to multiple hardware structural parameters, including the camera working distance *L*, focal length *f_p_*, the size of pixel *d*, and the angle *θ* between the camera’s optical axis and the light plane (observation angle). Additionally, the value of resolving power varies at different positions on the imaging plane of the camera. To further explore the influence of these structural parameters on measurement resolving power, this article simulates the relationship between each parameter and the resolving power, and plots the corresponding curves for illustration.

**Figure 4:**
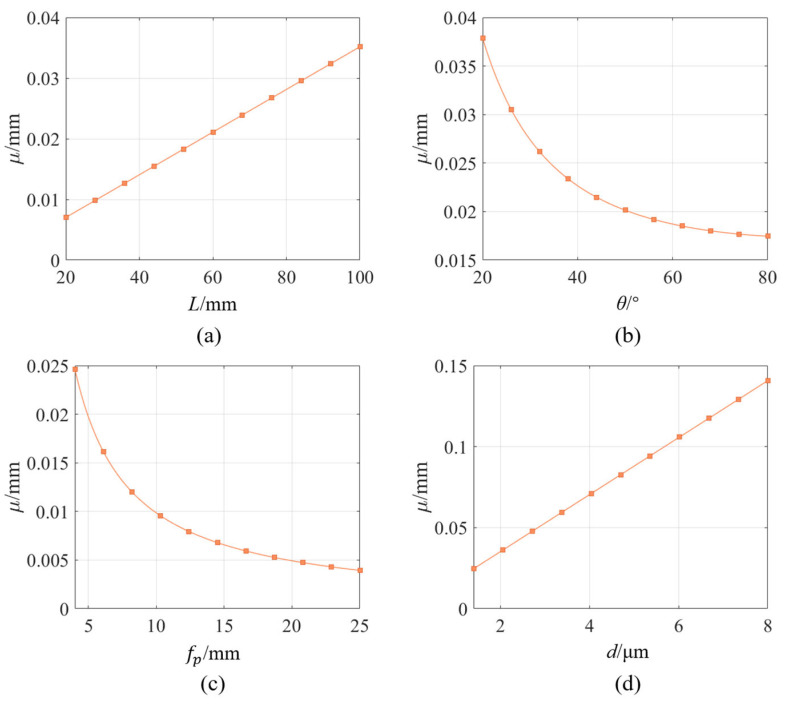
Influence of different parameters on measurement resolving power: (**a**) Working distance, (**b**) Observation angle, (**c**) Focal length, (**d**) Pixel size.

The preset sensor structure parameters in the simulation are as follows: working distance 70 mm, lens focal length 4 mm, observation angle 35°, pixel size 1.4 μm, and camera target resolution 2592 × 1944. From Equation (4), it can be noted that the measurement resolving power at different pixel positions in the camera imaging plane is different, but their variation with the structural parameters is uniform. Therefore, in the simulation, the measurement resolving power at the center point of the imaging plane is taken as an example for analysis, while only modifying one single variable parameter at a time. Figure 4 illustrates the influence of different parameters on the resolving power of the line-structured light system. It can be seen that the resolving power is proportional to the working distance *L* and the pixel size *d*, and inversely proportional to the observation angle *θ* and the focal length *f_p_*. According to the definition of resolving power, an increase in resolving power means that a one-pixel change in the imaging plane corresponds to a larger displacement change in real space. This can lead to significant positional deviations in the three-dimensional space due to minor errors in extracting pixel coordinates from images, which degrade the system’s measurement accuracy. Therefore, when designing a line-structured light measurement system, it is necessary to comprehensively consider the influence of multiple structural parameters to achieve the optimal resolving power for the system.

### 3.2. Optimal Accuracy Structural Optimization Design

Equation (4) illustrates the structural parameters that influence the resolving power of the line-structured light measurement system. However, in practical engineering problems, there are still many constraints that limit the range of variation for these parameters. For the multi-sensor inner wall measurement system designed for spherical cavity parts in this article, there are primarily two constraints. One of them is the limitation of the aperture for the parts. To ensure that the sensor can penetrate into the part and avoid collision risks during measurement, the sensor size should not be too large. On the other hand, the measurement system adopts multiple sub-sensors, requiring overlapping FOV between adjacent sub-sensors to ensure complete 3D measurement of the inner wall. When improving measurement accuracy by increasing the observation angle and reducing the sensor working distance, the geometric size of the sensor increases and the sensor’s FOV decreases, resulting in gaps in the 3D data measured by the system. The influence of these structural parameters and constraints on the system’s measurement accuracy is contradictory. Therefore, it is necessary to find the optimal solution for the system’s structural parameters while considering the limitations imposed by the constraints.

The measurement system designed in this article adopts four independent cameras, each paired with a light plane to form sub-sensors, collectively performing scanning measurements on the inner wall of the spherical cavity part. Due to hardware limitations, the parameters of the cameras in the measurement system are fixed. Additionally, for the measurement of inner-wall-shaped parts, the geometric size of the sensor is a more critical requirement in practical engineering applications, which will directly determine the feasibility of the measurement system. Therefore, the optimization design focuses more on the structural parameters that are more sensitive to geometric constraints, namely the camera’s observation angle and working distance.

**Figure 5:**
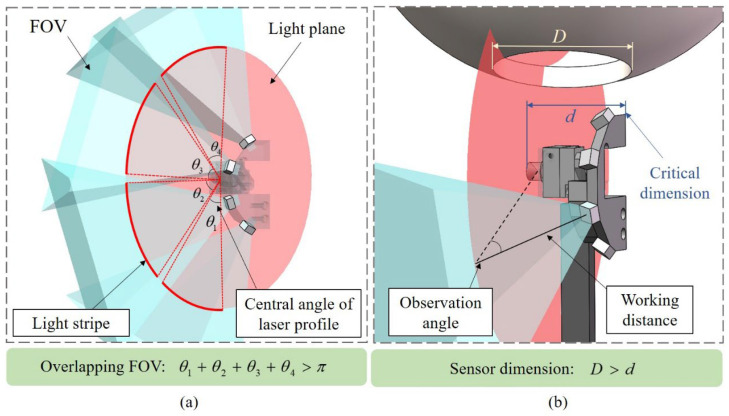
Constraints in measuring spherical cavity parts: (**a**) Overlapping FOV for adjacent sub-sensors, (**b**) Geometric dimensions of sensor constrained by parts.

Based on the above approach, the constraints in the measurement process are shown in Figure 5. The two constraints mentioned above are used to define the boundary conditions in the optimization objective function. The first constraint is to ensure that there is an overlapping FOV between the adjacent cameras, but this is difficult to calculate directly due to the differences in the poses of the cameras. Therefore, the measurement range of each sub-sensor is transformed into the central angle of the circular light stripe captured by each camera, as shown in Figure 5a. In the system, the light plane intersects with the inner wall of the spherical cavity to form a circular stripe, and the four cameras are uniformly distributed within a 180° range. Considering the symmetry of the measured part, it is only necessary to ensure that the central angle of the light stripe captured by a single sub-sensor is greater than 45° to satisfy the condition. For the convenience of analysis, by neglecting the size of hardware components such as cameras and lasers in the system, the critical dimension affecting sensor penetration can be calculated using the geometric relationship between the camera’s working distance and observation angle. This dimension must be smaller than the aperture of the parts, as shown in Figure 5b.

(5) $$\left\{\begin{array}{l} \min\mu (\theta ,L)\\ s.t.\left\{\begin{array}{c} \alpha (\theta ,L)\geq \alpha_{0}\\ D_{sensor}(\theta ,L)\leq D_{0} \end{array}\right.\\ (\theta ,L)\in \Omega \end{array}\right.$$

Based on the above analysis, regarding the resolving power as the objective function, the expression for this optimization problem is shown in Equation (5). Ω represents the value range of *θ* and *L*. α represents the central angle of the light stripe captured by the sensor. *D_sensor_* represents the critical dimension of the sensor. In the boundary conditions of the sensor, α_0_ and *D*_0_ denote the central angle of the light stripe and the spatial dimension constraints of the sensor, respectively. According to the expression of optimization, a penalty function approach can be used to construct the optimization objective function as shown in Equation (6), where σ represents the penalty factor, which is a sufficiently large positive number that forces the optimization objective function to achieve the optimal value while satisfying the constraints.

(6) $$\left\{\begin{array}{c} p_{1}=\min(\alpha (\theta ,L)-\alpha_{0},0)\\ p_{2}=\min(D_{0}-D(\theta ,L),0)\\ E=\arg\min\sqrt{\mu {\left(\theta ,L\right)}^{2}+{\sigma p_{1}}^{2}+{\sigma p_{2}}^{2}},\sigma >0 \end{array}\right.$$

## 4. Calibration of Multi-Sensor Measurement System

In this study, a multi-sensor measurement system is developed based on the line-structured light measurement principle. It is necessary to calibrate various parameters in the measurement model of the system to achieve high-precision measurement of the parts. The calibration procedure comprises three main steps. The first step is to calibrate the intrinsic parameters of the camera and the light plane of each sub-sensor. The second step is to calibrate the rotation axis parameters of the system. Using the initial relative position between each sensor and the rotation axis as designed, an optimization approach is applied by scanning standard ring gauges at multiple positions to calibrate the rotation axis parameters in the self-rotating system. The final step is to calibrate the relative position of each sub-sensor by using the rotation axis and light plane as constraints. By collecting the contour features of the ring gauge at multiple different positions, the 3D data obtained by each sensor in the measurement system can be unified. This section will introduce the axis calibration method in the self-rotating system and the multi-sensor calibration method, respectively.

### 4.1. Self-Rotating Axis Calibration

**Figure 6:**
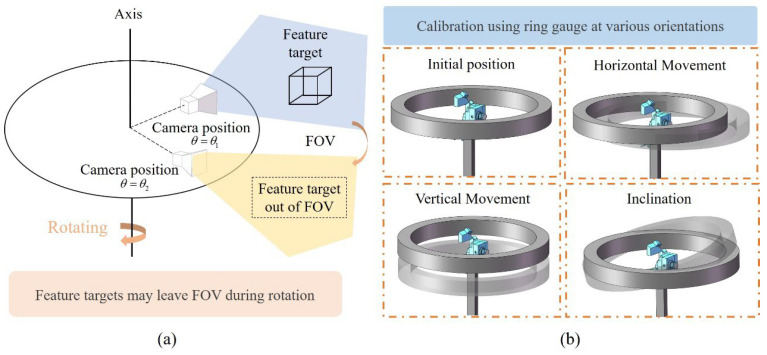
Challenges in calibrating the rotation axis of the self-rotating system and the calibration method: (**a**) Targets out of view during sensor rotation, (**b**) Calibration method based on ring gauge features.

The measurement system utilizes a self-rotating sensor to conduct scanning measurement on the inner wall of the part. Due to the continuous change in the camera’s FOV during rotation, it is difficult to determine the rotation axis position in the sensor coordinate system by constructing feature targets, as shown in Figure 6a. Therefore, a standard ring gauge with full circumferential features is considered for calibrating the rotation axis parameters. In this article, a two-point representation is used to describe the position and orientation of the rotation axis. The position of the rotation axis is determined by two points, *P*_1_(*x*_1_, *y*_1_, *z*_1_) and *P*_2_(*x*_2_, *y*_2_, *z*_2_). The direction of the rotation axis is determined by the vector $\vec{P_{1}P_{2}}$. The calibration procedure is shown in Figure 6b. A ring gauge with radius R is placed in different poses, and the sensor conducts rotational scanning measurements to acquire three-dimensional data. For any point (*X*, *Y*, *Z*) on the ring gauge, the 3D coordinate measured by the sensor is *P*(*x*, *y*, *z*). According to the system structure, Equation (7) can be obtained as follows.

(7) $$\left[\begin{array}{c} x\\ y\\ z\\ 1 \end{array}\right]=MR_{c}{\left(\theta \right)}^{-1}\left[\begin{array}{c} X\\ Y\\ Z\\ 1 \end{array}\right]$$

The matrix *M* represents the transformation matrix from the ring gauge coordinate system to the sensor coordinate system. To simplify calculations, it can be set as an identity matrix. The matrix *R_c_*(*θ*) represents the transformation matrix for rotating by angle *θ* around the rotation axis, which is determined by the axis parameters and the angle. It can be calculated using the Rodrigues formula.

By rotating the sensor with the turntable, multiple sets of 3D point clouds of the ring gauge are obtained. Each set of point clouds should form a standard cylinder with a radius equal to the nominal radius of the ring gauge in theory. Therefore, an optimization approach is used to search for the rotation axis parameters *P*_1_(*x*_1_, *y*_1_, *z*_1_) and *P*_2_(*x*_2_, *y*_2_, *z*_2_) to minimize the error of the cylindrical fitting of the ring gauge data calculated by *R_c_*(*θ*). Assuming *K* as a point on the axis of the fitted cylinder and *V* as the unit vector denoting the direction of the cylinder axis, the optimization objective function is presented in Equation (8).

(8) $$E=\arg\underset{P_{1},P_{2}}{\min}\sum_{i=1}^{N}{\left(\sqrt{{\left\|\vec{KP}\right\|}^{2}-{\left(\vec{KP}\cdot V\right)}^{2}}-R\right)}^{2}$$

### 4.2. Multi-Sensor Registration Based on Structural Constraints

The measurement system adopts multiple sub-sensors with low overlapping FOV for 3D measurement. To unify the coordinates of measurement data, it is necessary to calibrate the relative position between the sub-sensors. For the structural configuration of the measurement system, this article proposed a three-constraints calibration strategy based on the system’s structural features to calibrate the relative position between the sub-sensors. Within the system architecture, multiple sub-sensors are composed of individual cameras and a shared light plane. During the measurement process, the sensors synchronously perform rotational scanning under the drive of the turntable. Consequently, the rotation axis and light plane parameters within each sub-sensor’s coordinate system function as intrinsic structural constraints, facilitating the calculation of the relative position among the sub-sensors.

**Figure 7:**
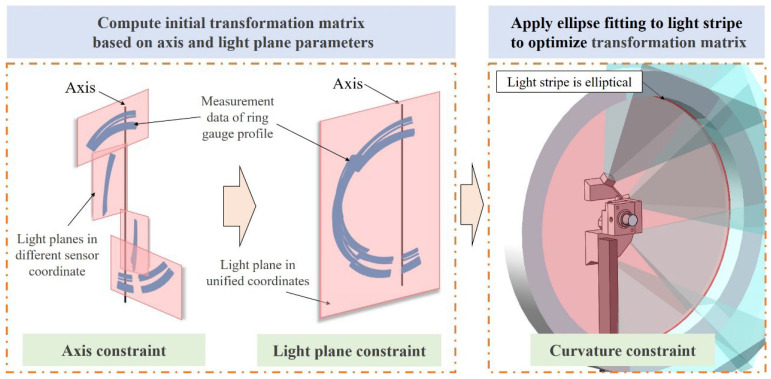
Three-constraints calibration method.

The multi-sensor registration method based on the structural constraints of the measurement system itself, proposed in this article, is illustrated in Figure 7. In addition to the system’s intrinsic structural features, ring gauges are used for registration during calibration. This method involves capturing 3D data of the ring gauge profiles at different positions and using the calibrated rotation axis and laser plane parameters to calculate the initial values of the transformation matrices between the sensors. Subsequently, the matrices are further optimized by fitting ellipses to each light stripe of the ring gauge. During the calibration process, the ring gauge is placed at different positions. At each position, all sub-sensors can acquire the contour data (*x_ij_*, *y_ij_*, *z_ij_*) of the ring gauge, where *i* represents the sensor index and *j* denotes the contour data at the *j*-th position. In the measurement system, the light plane intersects with the inner surface of the ring gauge to generate a light stripe. In theory, this stripe is a spatial ellipse, and the minor axis radius of the ellipse is equal to the nominal radius of the ring gauge. Therefore, an optimization approach can be considered to calculate the transformation matrices between the sensors. By performing ellipse fitting on the data obtained from each sub-sensor, the fitting error can be used as the optimization objective function to search for the optimal transformation matrix *R_i_*, *T_i_*, as shown in Equation (9).

(9) $$\left\{\begin{array}{c} E_{1}=\arg\underset{R_{i,}T_{i}}{\min}\sum_{i=1}^{4}\sum_{j=1}^{N}d_{plane}{\left(R_{i}\left[\begin{array}{c} x_{ij}\\ y_{ij}\\ z_{ij} \end{array}\right]+T_{i}\right)}^{2}\\ E_{2}=\arg\underset{R_{i,}T_{i}}{\min}\sum_{i=1}^{4}\sum_{j=1}^{N}d_{ellipse}{\left(R_{i}\left[\begin{array}{c} x_{ij}\\ y_{ij}\\ z_{ij} \end{array}\right]+T_{i}\right)}^{2} \end{array}\right.$$

In the equation, *d_plane_*(·) represents the distance from each point to the fitted plane after unifying the measurement data from all sensors. *d_ellipse_*(·) represents the difference between the sum of the distances from each point to the two foci and the major axis of the fitted ellipse.

## 5. Experiments

The experimental section consists of two main parts. The first part presents the optimization results of the system’s structural parameters and the calibration of the rotation axis in the self-rotating measurement system. The experimental results demonstrate the diameter deviation of the standard ring gauge measured by the sub-sensors and the comparison results with the existing methods, which prove the measurement accuracy and repeatability of the sub-sensors and highlight the effectiveness of the structural optimization design strategy and calibration method for the rotation axis. The second part introduces the multi-sensor calibration results based on the three-constraints method. The repeatability experiments on spherical cavity part measurements demonstrate the system’s measurement accuracy. The comparative experiments between the proposed method and traditional methods highlight the advantages and effectiveness of the proposed method.

### 5.1. Optimization Design of Structural Parameters and Rotation Axis Calibration

**Figure 8:**
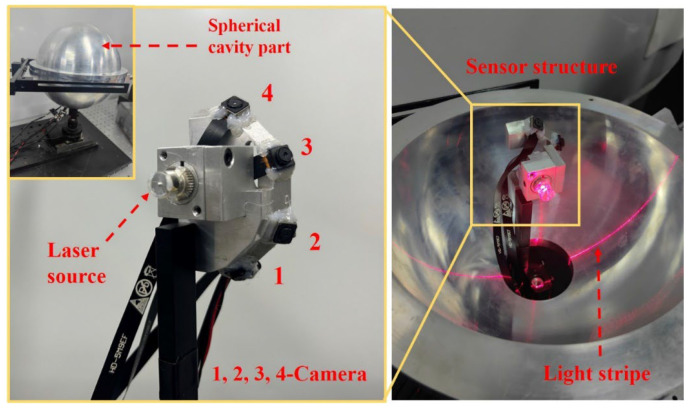
Physical system.

The experimental system built in this paper is shown in Figure 8. The measured object is a spherical cavity part composed of two hemispheres. Its radius measured by the coordinate measuring machine (CMM) is 100.008 mm, and the spherical cavity has a bottom opening with a diameter of 60 mm. The positioning accuracy of the turntable in the measurement system is 0.1°. Ignoring the dimension of the hardware, the working distance and observation angle of the camera in the system were calculated based on the structural optimization design method proposed in Section 3.2. Based on the preset parameter ranges *L* ∈ (50, 100) and *θ* ∈ (10, 80) according to the actual dimensions of the measured part, the optimal sensor structure has a camera working distance of 72.2595 mm and an observation angle of 38.2628°. Using the above results as a guide, the working distance of each camera in the system was set to 72 mm and the observation angle to 30° during actual operation to avoid the risk of collision between the system and the measured part. Since the light stripe captured by the camera is horizontally oriented and the imaging process ensures its placement near the central region of the image to maintain clarity, the measurement area is defined by points within the imaging plane where the vertical pixel coordinate *v* falls within the range (*v*_0_/2, 3*v*_0_/2). The average measurement resolving power of the pixels in this area is 0.0264 mm, and the maximum resolving power is 0.0429 mm. The design results of the parameters in the measurement system are shown in Table 1.

In the calibration of the measurement system, the camera and light plane parameters in the system were calibrated using Zhang’s method [33]. By placing a checkerboard at different positions intersecting with the light plane, a set of checkerboard images and images of light stripes projected on the checkerboard were recorded at each position. Because the 3D coordinates of the light stripe on the checkerboard can be calculated by the homography matrix, plane fitting was performed on the light stripes at all positions to achieve calibration of the light plane surface. The checkerboard is 12 × 9 with 5 mm spacing and ±10 μm accuracy. The calibration results of sub-sensors are in Table 2.

**Figure 9:**
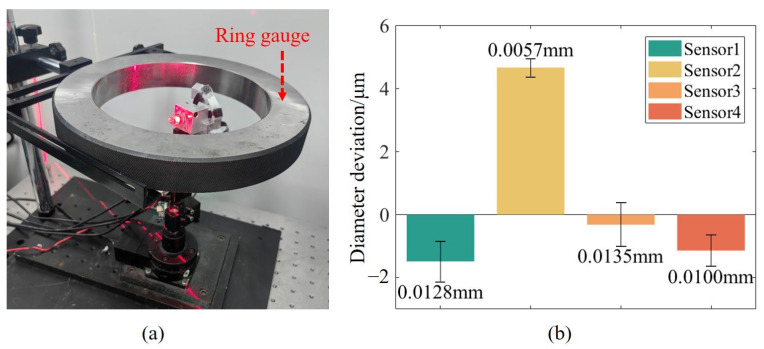
Rotation axis calibration results. (**a**) Physical system of axis calibration, (**b**) Measurement results of ring gauge diameter deviations by each sub-sensor.

Subsequently, the rotation axis of each sub-sensor was calibrated. Based on the optimization approach, the optimal rotation axis parameters in the sub-sensor coordinate system are obtained by scanning the ring gauge and performing cylindrical fitting. The position of the ring gauge in the experiment is shown in Figure 9a. To avoid the calculation results falling into a local optimum, the ring gauge positions need to be distributed around the rotation axis. In the calibration experiment, the ring gauge was placed at 8 positions with different postures. Because the relative position between sub-sensors and the rotation axis is different, two ring gauges with different diameters are used for calibration to ensure that the size of the ring gauge is suitable for the measurement of the FOV of different sub-sensors. The diameters of the ring gauges are 79.999 mm and 169.999 mm. To verify the effectiveness of the proposed rotation axis calibration method, each sub-sensor measures the ring gauge at eight positions different from those used during calibration. The diameter deviations of the ring gauge measured by each sub-sensor are shown in Figure 9b. In the results, the average diameter deviation is 5 μm, and the maximum standard deviation of the diameter deviation is 0.0135 mm, which is consistent with the theoretical measurement accuracy calculated in the sensor structural design.

**Figure 10:**
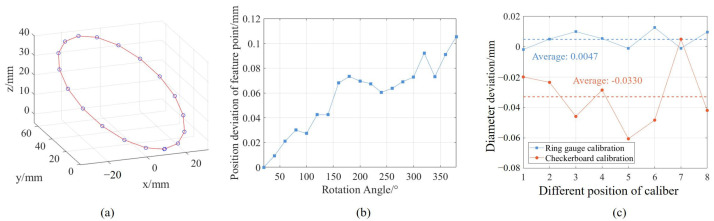
Comparative experiment of rotation axis calibration. (**a**) The spatial position of the camera origin during rotation, (**b**) Distance between the theoretical position and actual position of the camera origin at different rotation angles, (**c**) Comparative experimental results.

To verify the accuracy of the proposed method, sub-sensor 2 is taken as an example to make a comparison between the proposed method and the method in [22]. The experiment uses a checkerboard as a feature to calculate the pose changes of the camera before and after rotation. Based on the transfer process of calculating the sensor position in [22], a series of 3D coordinates of the camera origin during the rotation process were obtained, as shown in Figure 10a. These points were used as the feature points required for fitting the rotation axis, and the parameters were calculated by fitting a space circle to these points. However, when employing this method to calculate the position change of the camera during rotation, the current position of the camera is determined based on its previous position. This sequential dependency introduces cumulative errors in the camera’s positional tracking, which causes deviations in the calculation of the parameters of the rotation axis. Figure 10b shows the distance deviation between the theoretical and the actual positions of the camera origin during the rotation calibration process. Due to the influence of the cumulative error, the distance deviation increases with the increase of the rotation angle. The comparative experimental results of these two axis calibration methods on the diameter deviation of the ring gauge are shown in Figure 10c. The average measurement deviation of the ring gauge diameter by the proposed method is 0.0047 mm, and the standard deviation is 0.0057 mm, while the average measurement deviation of the ring gauge diameter by the comparison method is −0.0330 mm, and the standard deviation is 0.0205 mm. In the process of calibrating the rotation axis, the measurement points obtained by each set of ring gauges in the proposed method have consistent geometric characteristics, and the proposed method performs global optimization of the rotation axis, which makes the calibration results more accurate. The experiment results show that the proposed method in this article exhibits smaller diameter deviations and the fluctuation range is significantly reduced, which demonstrates the accuracy of the proposed method.

### 5.2. Multi-Sensor Calibration Experiments

**Figure 11:**
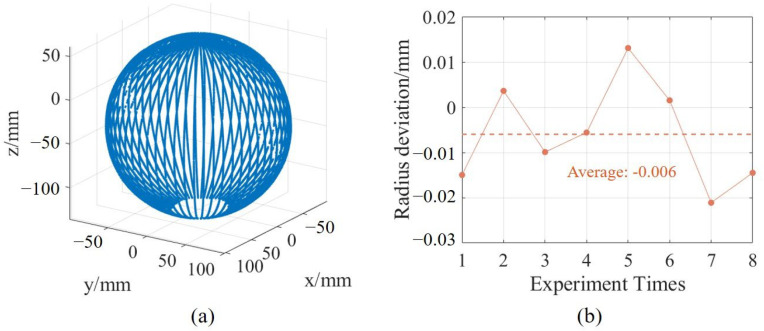
Measurement results of spherical cavity and repeatability. (**a**) Measurement point cloud of spherical cavity, (**b**) Radius deviation results.

After the rotation axis calibration, each sub-sensor in the system can perform 3D measurements through rotary scanning. After that, the relative position between each sub-sensor is calibrated by using the three-constraint method. The measurement system, after calibration, was used to measure the spherical cavity part, and the point cloud is shown in Figure 11a. To verify the repeatability of the measurement system, 8 repeated measurements were conducted on the spherical cavity part with radius deviations analyzed against CMM results, as shown in Figure 11b. The average deviation is −0.006 mm and the standard deviation of 0.0114 mm, which aligns with the repeatability of the ring gauge measurements, demonstrating the accuracy of the multi-sensor registration algorithm.

**Figure 12:**
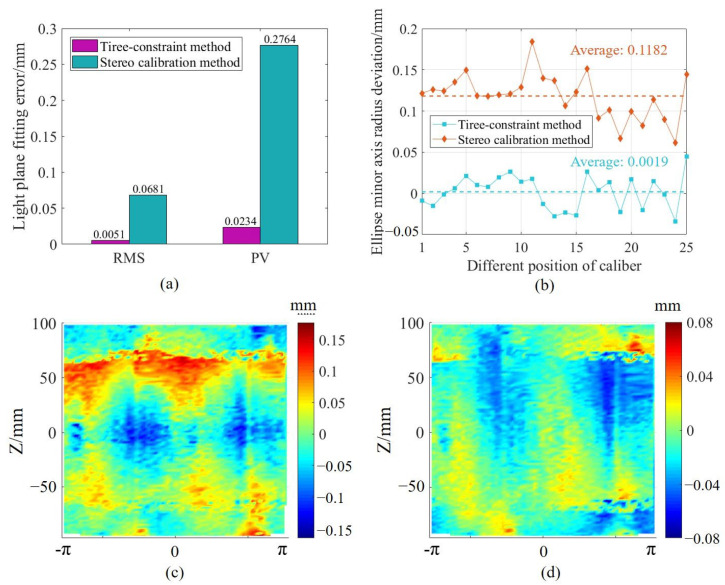
Comparative experimental results of multi-sensor registration: (**a**) Light plane fitting deviation, (**b**) Minor axis radius deviations in ellipse fitting, (**c**) Measurement error of spherical cavity after calibration using traditional method displayed in cylindrical coordinates, (**d**) Measurement error of spherical cavity after calibration using three-constraint method displayed in cylindrical coordinates.

To verify the effectiveness of the proposed multi-camera registration method, a comparative experiment was conducted with the traditional stereo vision calibration method [34]. Due to the low overlapping FOV between adjacent sub-sensors, a larger checkerboard target was used, and the cameras in adjacent sub-sensors were controlled to respectively capture some corner points in the checkerboard, which were used as features for registration. Since there are certain structural features in the measurement system, it was necessary to compare the registration results of different methods under these constraints in the experiment. In the experiments, a comparative analysis was conducted on multiple sets of ring gauge profile data acquired by the sub-sensors. Figure 12a shows the deviations of the registered data from both methods after plane fitting. In theory, the data obtained by the sub-sensors should lie on the same light plane. It can be seen that the peak to valley (PV) and standard deviation of the registered data obtained using the proposed method are significantly smaller than those obtained using the traditional method. Figure 12b shows the deviation in the minor axis radius of the fitted ellipse under the ring gauge constraint for both methods. The average deviation of the minor axis radius of the fitted ellipse obtained by the stere calibration method is 0.1182 mm, while the average deviation of the minor axis radius of the fitted ellipse obtained by the proposed method is only 0.0019 mm.

To further demonstrate the surface continuity of the proposed multi-camera registration method in processing point clouds, this paper chooses one set of results from repeatability measurements of a spherical cavity part to compare the error distributions of point clouds generated by the two approaches. Figure 12c,d demonstrate the error distributions of the measurement of the spherical cavity after the system calibration using the stereo vision method and the three-constraint method, respectively. The measurement results of the spherical cavity point cloud are mapped into the cylindrical coordinate system for display. In the results, the system calibrated by the stereo vision method measures the spherical cavity radius as 99.9493 mm with a standard deviation of 0.0665 mm. The system calibrated using the three-constraint method measures the radius as 100.015 mm with a standard deviation of 0.0197 mm, which is significantly smaller. Additionally, by comparing Figure 12c,d, it can be seen that the error distribution from the traditional calibration method exhibits noticeable discontinuities, whereas the error distribution from the three-constraint method calibration is smoother, demonstrating the effectiveness of the proposed multi-sensor matching method.

## 6. Discussion

This paper proposes a multi-sensor self-rotating scanning measurement system based on the principle of line-structured light, which has achieved 3D measurement of the inner wall of spherical cavity parts. Using a spherical cavity part with a diameter of 200 mm and a bottom aperture diameter of 60 mm as the experimental object, the measurement system can obtain the complete 3D point clouds of its inner surface through a rotary scanning method and achieve high-precision diameter measurements. For the measurement system developed in this study, its current limitation lies in the fact that it is only applicable to spherical cavity parts with dimensions similar to those of the experimental specimen. However, since the proposed system design and calibration method are universal, they can be adapted to components with different apertures by adjusting the existing structure while following the same system design strategy. Additionally, the current system does not fully consider the impact of potential measurement blind zones caused by complex inner-wall structures. Future research could incorporate this consideration into the theoretical framework of system design strategy to enhance its practical applicability.

The proposed method in this article emphasizes more on the feasibility of the algorithm from the perspective of external sensor parameters during system calibration. However, the influences of calibration errors from the intrinsic parameters of the sensor, motion errors of the mechanical structure, and the manufacturing errors of the system have not been modeled. In this study, the parameters of the measurement system were optimized through a calibration method to minimize the influence of errors. Although the current results do not directly model and analyze manufacturing errors, the theoretical values combined with calibration optimization can still provide a reference for industrial design. Subsequent research needs to reduce this error as much as possible through the improvement of algorithms and processing methods to ensure that the actual measurement accuracy of the system is consistent with the theoretical design.

## 7. Conclusions

Aiming at the problem of full-profile measurement of the inner-wall-shaped parts with a narrow aperture and large cavity, this paper proposes a structural design strategy and system calibration method for a line-structured light multi-sensor measurement system. In this system, the traditional linear scanning method is replaced by rotary scanning to achieve low-cost and high-precision measurement. To address the issue of significant cumulative errors in existing rotation axis calibration methods for a self-rotating system, an optimization-based rotation axis calibration method is proposed. Considering the structural constraints imposed by the unique features of the measured parts, a structural optimization algorithm is introduced to ensure that the sub-sensors achieve optimal theoretical measurement accuracy while satisfying the geometric constraints of the measured parts. To address the challenges of point cloud registration with low overlapping FOV, a multi-sensor registration method based on the structural constraints of the measurement system itself is proposed. This method eliminates the need for high-precision auxiliary equipment or complex calibration targets and achieves high-precision data fusion at a low cost, which offers a novel solution to the multi-sensor registration problem under low overlapping FOV. The experimental results on spherical cavity part measurements demonstrate the measurement accuracy and repeatability of the system proposed in this article. The realization of full-profile measurement for inner-wall-shaped parts with narrow-aperture and large-cavity verifies the feasibility of the proposed algorithm.

## Figures and Tables

**Table 1:** Results of parameter design for the measurement system.

Parameters	Value
Working distance *L*	72 mm
Observation angle *θ*	30°
Focal length *f*	4 mm
Pixel size *d*	1.4 μm
Camera horizontal resolution	2592
Camera vertical resolution	1944
Horizontal field *α*	59°
Vertical field *β*	43°

**Table 2:** Calibration results of the camera and light plane in sub-sensors.

Number	Reprojection Error	Standard Deviation of Light Plane
1	0.27 pixel	0.0178 mm
2	0.20 pixel	0.0150 mm
3	0.26 pixel	0.0189 mm
4	0.22 pixel	0.0170 mm

## Data Availability

The data are not publicly available because the data also form part of an ongoing study.
